# Development and feasibility of a function-based preventive intervention for lifestyle-related disorders

**DOI:** 10.1186/s12889-024-18017-8

**Published:** 2024-03-04

**Authors:** Lena Bornhöft, Daniel Arvidsson, Anna Bergenheim, Mats Börjesson, Jonatan Fridolfsson, Margareta Hellgren, Lena Nordeman, Maria EH Larsson

**Affiliations:** 1 Research, Education, Development, Innovation and Implementation, Primary Health Care,; 2 Unit of Physiotherapy, Department of Health and Rehabilitation, Institute of Neuroscience and Physiology, Sahlgrenska Academy at University of Gothenburg, Gothenburg, Sweden; 3 Primary Care Rehabilitation, Närhälsan Torslanda Rehabilitation Clinic, Gothenburg, Region Västra Götaland Sweden; 4 Center for Health and Performance, Department of Food and Nutrition and Sport Science, Faculty of Education, University of Gothenburg, Gothenburg, Sweden; 5 Department of Molecular and Clinical Medicine, Sahlgrenska Academy at University of Gothenburg, Gothenburg, Sweden; 6 Center for Lifestyle Intervention, Department of MGAÖ, Sahlgrenska University Hospital, Gothenburg, Region Västra Götaland Sweden; 7 General practice - Family medicine, School of Public Health and Community Medicine, Institute of Medicine, Sahlgrenska Academy, University of Gothenburg, Gothenburg, Sweden; 8 The Skaraborg Institute, Skövde, Sweden

**Keywords:** Lifestyle-related disorders, Prevention, Physical activity, Function, Risk profile

## Abstract

**Background:**

The enormous effect of lifestyle-related disorders on health of the global population warrants the development of preventive interventions. Focusing on musculoskeletal health and physical activity may be a way to encourage necessary lifestyle changes by making them more concrete and understandable. The aims of the current study were to develop a function-based preventive intervention aimed at lifestyle-related disorders in physically inactive 40-year-old people and to investigate the feasibility of the intervention. The feasibility study aimed to solve practical and logistical challenges and to develop the intervention based on the experiences of participants and involved clinical personnel according to defined criteria.

**Methods:**

Development of the standardised functional examination was based on literature-validated tests and clinical reasoning. Development of a risk profile was based on the functional examination and similar profiles which have already proved feasible. The feasibility of the functional examination and risk profile, together with function-based lifestyle counselling was tested on 27 participants in a pilot study with two physiotherapist examinations over a four-month period. Practical results and feedback from participants and collaborating personnel were examined.

**Results:**

The functional examination consists of 20 established tests not requiring specialised equipment or training which were deemed relevant for a middle-aged population and a sub-maximal ergometer test. The risk profile consists of seven functional dimensions: cardiovascular fitness, strength in upper extremity, lower extremity and trunk, mobility, balance and posture, and three non-functional dimensions: weight, self-assessed physical activity and pain. Each dimension contains at least two measures. The participants appreciated the intervention and found it motivating for making lifestyle changes. They found the tests and risk profile understandable and could see them as tools to help achieve concrete goals. The examination required 60–75 min for one physiotherapist. The recruitment rate was low and recruited participants were highly motivated to making lifestyle changes.

**Conclusion:**

This project developed a functional test battery and risk profile aimed at inactive 40-year-olds which fulfilled our feasibility criteria. Functional screening and lifestyle counselling were found to be of value to a sub-group of inactive 40-year-olds who were already motivated to improve their health situations.

**Trial registration:**

ClinicalTrials.gov: NCT05535296 first posted on 10/09/2022.

**Supplementary Information:**

The online version contains supplementary material available at 10.1186/s12889-024-18017-8.

Lifestyle factors influence the risk of morbidity and premature mortality, and low level of physical activity is associated with the development of lifestyle-related conditions such as cardiovascular and metabolic diseases, osteoarthritis and other musculoskeletal disorders [1]. Physical inactivity levels continue to rise, primarily in high-income countries [2]. This causes immense quality of life impacts including reduced life expectancy and increased levels of illness and disability and equally enormous resource demands [3, 4]. Increased knowledge about the types of preventive interventions that most efficiently influence lifestyle changes in health-promoting directions is needed. This knowledge can then be used to better tailor treatment and advice to patient needs and to individual risk factors.

Preventable lifestyle-related disorders (LRD), including cardiovascular, metabolic and musculoskeletal health problems, account for up to 40% of visits within primary care [5]. Early management strategies that give the patient tools to make lifestyle choices which may prevent later development of health conditions requiring medical treatment could reduce suffering for many patients, relieve the pressure on healthcare, and make resources available for patients with other health problems.

Research and clinical observations indicate that many patients have an inadequate understanding of their own physical capacity and that many are insufficiently motivated to make necessary lifestyle improvements [6]. Healthcare providers seldom have time for more than general lifestyle advice nor detailed knowledge about the patient’s functional capacity or lifestyle habits. If high risk patients were detected in time, perhaps development of both pain and disease could be avoided. Lack of time, the high demand for healthcare visits and insecurity in own capacity to provide suitable lifestyle counselling may contribute to a lack of preventive advice from caregivers or to general preventive advice which may or may not be sufficiently motivating for the individual patient [6].

Primary prevention programs in general seem to have good health effects. One systematic review found many primary prevention programs for cardiovascular diseases and metabolic syndrome to have positive effects on health and in some cases also proved to be cost-effective [7]. The Västerbotten Intervention Programme (VIP) in northern Sweden has been associated with a reduction of cardiovascular disease and associated early mortality by combining medical examinations with person-centred lifestyle advice [8]. However, there are pertinent aspects of health and well-being that this program does not address, such as functional capacity.

Physical activity level, physical function and weight all influence incidence and intensity of lifestyle-related illness [9]. Musculoskeletal pain can be involved as it is associated with avoidance of or limiting physical activity which in turn leads to worse function and increased risk for obesity and metabolic and cardiovascular diseases [8, 10]. Previous knowledge is limited of how testing physical function and giving related feedback affects health outcomes and lifestyle factors, such as physical activity level, over time. An American study showed that people experience such examinations positively and see value in such testing [11]. Generally, functional examinations and testing by a physiotherapist are unusual before symptoms become apparent. Lifestyle choices made early on in life and often unconsciously may have far-reaching and long-term effects on later health of which the individual may be unaware, with symptoms of ill-health becoming apparent only in middle-age or later [12].

Low physical activity is a major risk factor for all lifestyle-related disorders and is possible to influence in both the short- and long-term [13]. The Swedish programme Physical Activity on Prescription (PAP), which supports increased general physical activity and focuses primarily on secondary prevention among patients with metabolic syndrome, has been shown to lead to increased physical activity and improved physical function [14]. As many lifestyle-related disorders develop slowly, it is reasonable to use physical activity level to monitor lifestyle change and risk for LRD. Both subjectively and objectively measured physical activity with accelerometers give important information for following an individual’s physical activity level over time [15].

According to the Health Belief Model, health behaviour may be influenced when personal beliefs and knowledge about health risks [14] and methods for reducing them are targeted in preventive strategies [16, 17]. A person-centred approach to maintaining health, based on motivational interviewing, should lead to more positive results than general information [18]. Motivational interviewing has shown promise in changing health-related behaviours [18–20] and in increasing adherence to exercise programs [21]. Those people who run measurable risks for specific conditions may do so because their own previous lifestyle choices have been sub-optimal. In order to make well-informed lifestyle decisions, people may need to understand their own health conditions in greater detail. Those who need to make lifestyle changes to maintain or increase health may also need specific guidance to accomplish their goals [18].

It is possible that physical function and physical activity level can be used to monitor and influence risk for LRD. Monitoring requires broad screening of the population. Influencing requires that people have good understanding of their own health and lifestyle situations and that those who need support for making changes have access to it.

Ageing increases risk of LRD. It is unknown which timepoint is the most optimal to achieve reasonable preventive effects. Implementation of the VIP program in northern Sweden has, in several regions, focused on 40-year-olds. We continue this focus in this study assuming that, at the age of 40, many people may have an increased interest for maintaining and improving health while relatively few have developed manifest LRD. In addition, the prevalence of metabolic syndrome starts to increase more sharply around the age of 40 [22].

The aims of the current study were to develop a function-based preventive intervention aimed at lifestyle-related disorders in physically inactive 40-year-old people and to investigate the feasibility of the intervention. The feasibility study aimed to solve practical and logistical challenges and to develop the intervention based on the experiences of participants and involved clinical personnel.

## Methods

This project contains three main sections: development of a functional examination protocol, development of a risk profile, and a feasibility investigation of an intervention where inactive 40-year-old people were examined and received feedback according to the functional examination protocol and risk profile.

### Functional examination protocol

A literature search was performed primarily in the medical database PubMed concerning established functional tests. Known tests and tests which were new to the research group were appraised for the dimensions: fitness, strength, mobility, balance, and posture. Clinical reasoning within the research group was used to evaluate relevance for each test for inactive middle-aged people. In order to be relevant to Swedish primary care physiotherapists, who are the intended users of this examination protocol, tests which do not require specialised training or equipment, which were simple, quick, easy to instruct and to perform were prioritised. The diversity of physical dimensions and tests of interest and the judgemental requirements for inclusion precluded a strict systematic literature review. However, the evidence for each proposed test was examined concerning reliability and validity and published normal or recommended values applicable to a 40-year-old population.

### Risk profile

The risk profile used in the VIP program in northern Sweden was used as a visual base for the risk profile in this project [23]. Current dimensions of the VIP risk profile were examined for relevance to this project. However, instead of dimensions such as blood pressure and lipid levels, the functional dimensions described above were used. Functional dimensions were chosen based on clinical reasoning, considering outcomes which were deemed to be objectively measurable by physiotherapists and possible to influence by participant lifestyle decisions. The functional tests chosen after the literature search were grouped in relevant dimensions of physical function. At least two tests were grouped together to allow for a more comprehensive understanding of each particular dimension. Ranges and cut-off levels were calculated for each test and a system for calculating risk levels for each dimension was developed aiming at consistency and transparency throughout the profile.

### Feasibility investigation

The feasibility investigation included functional examinations, standard medical examinations, objective measurement of physical activity, and feedback with test results and lifestyle counselling. Contact information to 40-year-old people living a geographic region in western Sweden with areas with mixed socioeconomic conditions was obtained from Statistics Sweden (Statistics Sweden (scb.se)). Invitation letters were sent out aiming at recruiting approximately 25–30 people. Interested recipients of the invitation letters contacted the research team. Presumptive participants were screened by telephone according to inclusion and exclusion criteria.

*Inclusion criteria*: 40 years of age during the study year; self-assessed as physically inactive, as inactive people stand to gain the most from the intervention; live near enough examination location to attend visits; have relatively normal general mobility (can walk without support and can use all four extremities without self-assessed difficulties), to be able to perform the functional tests. *Exclusion criteria*: Self-reported regular moderate-to-intensive exercise more than once a week, as this can be interpreted as similar efforts to prevent lifestyle-related disorders as the intervention; severe mental illness or intellectual disability, as participants are expected to answer questionnaires, follow instructions and have the ability to make independent lifestyle decisions; pregnancy or ongoing hospital-based treatment, to increase probability that baseline values represent the person’s usual health state and functional capacity.

*Procedure*: Participants answered questionnaires regarding demographics, health and lifestyle through the Research Electronic Data Capture (REDCap) platform [24, 25] (Table 1). Well-established questionnaires within research studies with good evidence for measuring the desired quality in similar populations were chosen to measure stress, anxiety, depression, health-related quality of life, risk for developing chronic pain, and physical activity. Mental health was examined as it affects overall well-being and might affect results of the intervention and need to be included in the statistical analysis of a larger study. Health-related quality of life is important as lifestyle interventions affect many aspects of participants' lives. Functional level may have a significant effect on the risk for developing chronic pain and high-risk patients may need more support to make lifestyle changes. Physical activity level is clearly an area of interest and is included in the risk profile. No published questionnaire was found to measure self-assessed motivation for lifestyle change to improve health or for self-assessed physical function according to the dimensions which were to be examined in this study. Project-specific questions were, therefore, also posed to enable comparisons between objectively measured and subjectively assessed function. The questionnaire for smoking habits is based on regional healthcare routines. Participants' physical activity levels were measured objectively for one week with accelerometers. They were examined by a nurse who measured height, body weight, waist circumference and blood pressure and prescribed the following blood tests: glucose, total cholesterol, triglycerides, low density lipoproteins, high density lipoproteins. All participants were also examined by a physiotherapist who performed tests of fitness, strength, mobility, balance, and posture according to the functional examination protocol. All participants received feedback from the nurse regarding the first part of the examination. In addition, approximately half the group received feedback from a second physiotherapist regarding the functional test results in a motivational interview [19]. Results from the functional examination were compiled in a risk profile to illustrate risk levels associated with individual parameters or relation to population norms. Participants were supported in setting goals for desired change and in making realistic plans to achieve these goals. Follow-up examinations were made at 3–4 months according to the same procedure as above and participants wore accelerometers for one week once again. All participants received feedback from all examinations at follow-up. The goals and plans made in the group who had initially received lifestyle counselling were also followed up. Clinical investigators and participants were blinded as to the accelerometer results until after all examinations were complete.

**Table 1:** Questionnaires used at inclusion and follow-up

Questionnaire	Topic	References
Stress and Crisis Inventory-93 (SCI-93)	Mental health	[27]
Hospital Anxiety and Depression Scale (HADS)	Mental health	[28]
Saltin-Grimby Physical Activity Level Scale	Self-assessed physical activity	[29–31]
NBHW Physical Activity	Self-assessed physical activity	[32, 33]
SED-GIH	Sedentary behaviour	[34]
Euroqol 5 dimensions-3 L	Health-related quality of life	[35]
Örebro Musculoskeletal Pain Screening Questionnaire– short form	Risk for developing chronic pain and for pain-related sickness absence	[36–38]
Smoking habits*	Smoking habits	
Project-specific questionnaires*	Motivation for change Self-assessed function	

NBHW=(Swedish) National Board of Health and Welfare; *see Additional file 1

#### Feasibility outcomes

Exertion levels for each of the functional tests were graded at inclusion on the Borg Rating of Perceived Exertion Scale (min-max 6–20) [38]. Participants also graded how easily understandable the instructions for each test were on a 5-point numerical rating scale (NRS). Project-specific evaluations were completed after follow-up concerning impressions of the intervention and including overall questions concerning exertion and understandability. Four participants were interviewed in a focus group concerning their experiences and opinions. Focus group participants were recruited based on group allocation, sex, expressed interest in the project, language ability, and availability for the proposed interview time. Participants were contacted after all the examinations and counselling sessions until four were recruited to the focus group. A 40-minute interview was held digitally through Microsoft Teams and was led by a researcher not involved in the project. The interview was recorded, transcribed verbatim and summarised thematically. Spontaneous impressions concerning practicalities of the functional examination protocol expressed during the examinations were noted and evaluated continuously by the project management. Participating clinical personnel also completed project-specific evaluations of their roles and responsibilities in the project and were encouraged to propose solutions to difficulties as they arose throughout the study.

Feasibility would be determined based on participant and personnel evaluations and experiences, on time expenditure, exertion levels, understandability, and practicalities. To be considered feasible, the following was required:

- At least mean of 3 on a 5-point NRS for the participant evaluation question “How worthwhile in general was this intervention– examinations, risk profile, advice– for you and your state of health?”
- At least mean of 3 on a 5-point NRS for the personnel evaluation questionnaire.
- Mean time for the functional examination less than 90 min.
- Mean exertion level for the functional examination less than 13 on the Borg Scale of Perceived Exertion.
- At least mean of 3 on a 5-point NRS for understandability of test instructions.
- Solutions can be devised for any practical difficulties which arise.

Other participant experiences expressed on evaluation questionnaires and in a focus group interview would also be weighed in the overall assessment of feasibility.

Intervention outcomes included demographic and health variables, level of motivation for making necessary lifestyle changes to improve health, and self-assessments of functional capacity. Questionnaire, functional, accelerometric, medical examination and blood test results were collected as part of the intervention but are not included in the feasibility investigation.

## Results

### Functional examination protocol

A protocol for a standardised examination of functional outcomes was prepared based on literature-validated tests. In all, 21 functional tests were included in the analysis (Table 2). The Ekblom-Bak sub-maximal ergometer test and the 2-minute Step-test were chosen to examine cardiovascular fitness. Measures of strength in the upper extremity are based on Handgrip Strength using the Jamar hand dynamometer (Performance Health International LTD, Nottinghamshire, UK) and on a Biceps test. Strength in the lower extremity is measured with the 30-second Chair-stand test and Single-foot Heel Rises. Endurance in trunk muscles is measured with the Plank test, the Back Endurance test, and the Supine Bridge test. Mobility is examined with the Sit-Rise test, the Finger-Floor test, the Lateral Flexion test, and Beighton’s Hypermobility Score. Balance is examined with the Stand-on-one-leg eyes open/eyes closed tests, the Functional Reach and Lateral Reach tests, and the Sharpened Romberg test. Many postural measures are subjective and unvalidated and were not currently applicable in this context. Chosen for this project were the Occiput-to-Wall test, the Navicular Drop test and test of Patella Mobility when Standing. Details on the performance, rationale and references for each test can be found in Table 2.

**Table 2:** Included functional tests

Dimension/Test (references)	Rationale	Performance
**Fitness**		
Ekblom-Bak ergometer test [40, 41]	Established test. Takes less than 10 min. Normal values available according to age and sex. Validity tested. Sub-maximal test so should permit performance of other tests shortly afterwards.	Published manual: https://www.gih.se/ekblombaktest
2-minute Step-test [42–46]	Established test, simple, quick, minimal equipment. Normal values available for age ≥ 50. Extrapolated reference value. Values which predict later physical independence available. Validity and reliability tested.	March on the spot as fast as possible without running for 2 min. Lift knees to halfway between patella and SIAS. Count number of steps with right leg.
**Strength upper extremity**		
Handgrip Strength [47–52]	Established test, simple, quick, minimal equipment. Normal values available according to age and sex. Reliability tested for several target groups. Predicts cardiovascular disease and early mortality. Correlated to osteoporosis, general weakness, falls/fractures, diabetes, multimorbidity, dementia, depression, sleeping problems and quality of life.	Sit without arm or back support. Hold elbow at 90° and with a small gap between elbow and trunk. Grip a Jamar hand dynamometer as hard as possible for 3–5 s. Note maximum value. Repeat 3 times per hand. Alternate measurements of right and left so that each hand has at least 20 s to rest between measurements. Use maximum values for right and left.
Biceps [42–44]	Established test, simple, quick, minimal equipment. Normal values and values which predict later physical independence available for age ≥ 60. Extrapolated reference value. Validity and reliability tested among older adults.	Sit without arm or back support. Free choice of arm. Start with elbow in full extension and holding a dumbbell (2 kg for women, 4 kg for men). Perform as many full flexions of the elbow as possible during 30 s. Examiner holds two fingers on the biceps. Contact with participants underarm ensures full flexion.
**Strength lower extremity**		
30s Chair-stand [42, 43, 53, 54]	Established test, simple, quick, minimal equipment. Normal values available for younger and older age groups. Extrapolated reference value. Validity and reliability tested among older adults. Validity tested as fitness indicator among younger adults.	Sit on chair, height 45 cm, arms crossed over chest. Rise to full standing position with full extension in knees and hips as many times as possible during 30s.
Single-foot Heel Rises [55–57]	Established test, simple, quick, minimal equipment. Normal values available. Associated with knee osteoarthritis, and balance problems, fall risk and mobility impairments in elderly.	Stand on 1 foot on 10° wedge near a wall with balance support of 2 fingers on each hand on the wall. Lift heel as high as possible at the rate of 1 heel rise/s. Use a metronome to hold the pace. Maximum number of repetitions at same pace.
**Strength trunk muscles**		
Plank [58–60]	Established test, simple, quick, minimal equipment. Normal values available. Validity and reliability tested. Endurance in trunk muscles associated with back problems.	Lie on stomach. Lift body in a straight line resting on elbows and toes. Hold position as long as possible.
Back Endurance [61–64]	Established test, simple, quick, minimal equipment. Normal values available. Validity tested. Endurance in back muscles associated with back problems in men.	Lie prone on examination bench with SIAS in line with the edge and upper body free. Support with hands on a chair until test starts. Strap lower body to bench with one belt below the hips and one at ankle level. Lift hands and cross arms over chest. Hold upper body parallel with floor for as long as possible.
Supine Bridge [58, 60]	Established test, simple, quick, minimal equipment. Normal values available. Validity and reliability tested. Endurance in trunk muscles is associated with back problems.	Lie on back with bent knees. Feet on floor, hip breadth. Hands by ears or crossed over chest. Raise pelvis so that knees, hips and shoulders form a straight line. Hold as long as possible. If still holding at 2 min, extend the dominant* leg at the knee.
**Mobility**		
Sit-Rise [65–67]	Established test, simple, quick, minimal equipment. Normal values available. Correlates to general flexibility. Predicts early mortality.	Stand in front of an exercise mat. Sit down on the mat using as little support as possible. Stand up again with as little support as possible. Maximum 5 points for sitting and 5 points for rising, reduce by 1 point for every point of contact and by 0.5 points for uncontrolled movement. Best of 2 repetitions.
Finger-Floor [68–71]	Established test, simple, quick, minimal equipment. Values which predict low back pain and return to work after sick leave for low back pain available. Correlates to reduced back pain after treatment.	Stand with feet together, extended knees, no shoes. Bend forward as far as possible while keeping knees extended. Measure distance from tip of middle finger to floor.
Lateral Flexion [72–76]	Established test, simple, quick, minimal equipment. Limited lateral flexion predicts low back pain. Reliability tested. Cut-off values available.	Stand with heels and shoulders against a wall, 15 cm between heels. Arms hanging along sides. Measure from floor to tip of long finger. Bend to the side letting the hand glide down the leg. Measure distance from floor to tip of long finger. Mean of left and right.
Beighton Hypermobility Score [77, 78]	Established test, simple, quick, no equipment. General hypermobility associated with joint pain. Accepted system for grading degree of hypermobility.	1 point for each of the following: at least 90° extension in little finger, > 10° hyperextension in elbow or knee, can bend the wrist enough for the thumb to touch the underarm, standing with extended knees can place palms on floor. All tests made bilaterally except for the standing flexion.
**Balance**		
Stand-on-one-leg-eyes-open [79, 80]	Established test, simple, quick, minimal equipment. Normal values available but tested with maximum 2 min. Validity and reliability tested. Measures static balance.	Stand on one leg without support for as long as possible without moving the foot on the floor. Free choice of leg. Arms hanging at sides from start but arm movement may be used to maintain balance. Maximum 3 min.
Stand-on-one-leg-eyes-closed [80]	Established test, simple, quick, minimal equipment. Normal values available but tested with maximum 2 min. Validity tested. Measures static balance.	Stand on one leg with eyes closed without support for as long as possible without moving the foot on the floor. Free choice of leg. Arms hanging at sides from start but arm movement may be used to maintain balance. Maximum 3 min.
Functional Reach [81–86]	Established test, simple, quick, minimal equipment. Normal values available. Validity and reliability tested. Correlates to Bergs balance test and to fall risk. Measures dynamic balance in the sagittal plane.	Stand with right side towards a wall but without touching, wearing comfortable shoes. Stretch right arm forward to 90°. Mark starting point on the wall from the tip of the middle finger. Stretch as far forward as possible without moving the feet or touching the wall. Mark the furthest point from the tip of the long finger. Measure horizontal distance between the 2 points. Best of 2 tries.
Lateral Reach [82, 83, 87]	Established test, simple, quick, minimal equipment. Normal values available. Validity and reliability tested. Measures dynamic balance in the frontal plane.	Stand with back towards a wall but without touching, wearing comfortable shoes. Stretch one arm to the side to 90°. Mark starting point on the wall from the tip of the middle finger. Stretch as far to the side as possible without moving the feet, touching the wall, bending the knees or twisting the trunk. Mark the furthest point from the tip of the long finger. Measure horizontal distance between the 2 points. Repeat 3 times/side. Use mean values.
Sharpened Romberg [88, 89]	Established test, simple, quick, minimal equipment. Normal values available. Validity and reliability tested. Calculated reference value.	Stand heel to toe with the dominant foot forward. Arms crossed over the chest. Eyes closed. Time until eyes open, foot moves or support is taken with hands. Repeat 3 times. Use mean value. Maximum 3 min total for 3 repetitions.
**Posture**		
Occiput-to-wall [90]	Established test, simple, quick, minimal equipment. Normal values available. Related test, Tragus-to-wall, is validity and reliability tested and correlates to Occiput-to-wall. Correlates to radiological deviations and other mobility and postural measurements.	Stand with back towards a wall, heels 10 cm from the wall, wall contact with pelvic region and thoracic spine. Pull in chin and press back of head against the wall. Contact– yes or no.
Navicular Drop [91–93]	Established test, simple, quick, minimal equipment. Recommended values available. Validity and reliability tested. Measures pronation which is associated with low back pain, pain and injuries in the lower extremity and predicts ankle injuries in children who play sports.	Stand barefoot with most weight on one foot. Os navicularis is marked with a pen on the non-weightbearing foot. Examiner adjusts foot into neutral position and measures the distance between the floor and os navicularis. Redistribute weight to normal standing position. Measure distance from floor to os navicularis once more. Use difference between measurements for each foot.
Patella Mobility in Standing [94, 95]	Established test, simple, quick, no equipment. Patella is locked if knee is flexed or hyperextended. Hyperextension is associated with knee pain among the obese and predicts knee injuries among children who play sports.	Stand relaxed in habitual pose. Examiner tests patella mobility. Locked– yes or no.

SIAS = spina iliaca anterior superior; *dominant leg defined by preferred leg to kick a ball

### Risk profile

**Fig. 1:**
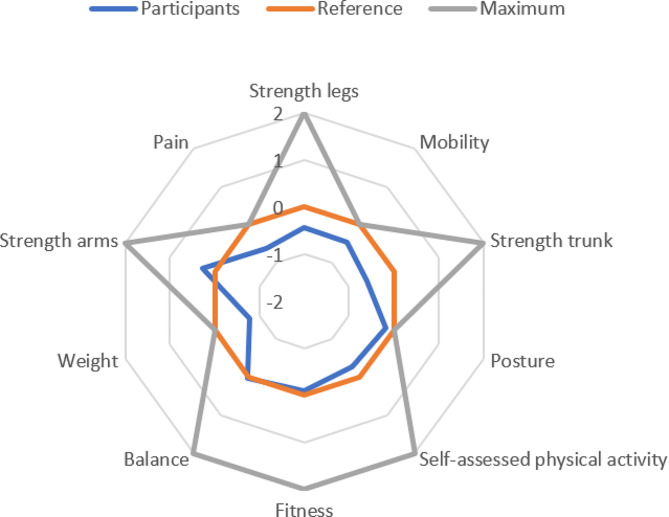
Participant values, reference values and maximum values on the risk profile

For the purpose of this study, we created a new risk profile which was visually based on the model used in the VIP program [23] but adapted to the functional outcomes examined in this project. Risk levels for ill-health were used when published values were available. Otherwise, population norms were used to describe low risk levels. Seven functional dimensions were included in the risk profile: fitness, strength in upper extremity, strength in lower extremity, strength in trunk muscles, mobility, balance, and posture. Three non-functional dimensions were also included: weight, self-assessed physical activity, and pain assessment. A list of normal or recommended values for 40-year-olds, sex-based values when relevant and available, was compiled. A point system where 0 points were given for each test for normal or recommended values ± 10%. In relation to this reference value, 1 or − 1 is given for values 10–30% from 0-level and 2 or − 2 for values more than 30% from 0-level. For Weight accepted cut-offs for normal weight, overweight/underweight and obesity were used. For the Beighton Hypermobility Score, Posture, Pain and two of the Self-assessed Physical Activity measurements, the possible results were given point values based on clinical reasoning and following the point pattern for the other dimensions as well as possible (Table 3). For the 2-minute Step-test, the 30-second Chair-stand test, the Biceps test and the Sharpened Romberg test, the 0-level for 40-year-olds was extrapolated from values for other age groups. The strength and fitness tests have differentiated normal values based on sex, as do Functional Reach, Sit-Rise, Lateral Flexion and Waist Circumference. Mean points for the tests belonging to each dimension are presented as the risk level for that dimension and are included in the risk profile (Fig. 1). For Mobility, Posture, Weight and Pain, the maximum point value attainable on the risk profile is 0. For the other dimensions, maximum is 2. For all dimensions except Posture, minimum is -2. For Posture, minimum is -1.

**Table 3:** Reference levels for the risk profile and risk level calculations

Test/Dimension	0-level ± 10%*		Risk level
	Male	Female	
**Fitness**			(F1 + F2)/2
F1: Ekblom-Bak cycle test (ml/kg/min)	39.3–42.7	32.8–35.9	
F2: 2-minute Step-test (n)	104–128	94–114	
**Strength upper extremity**			(SUE1 + SUE2)/2
SUE1: Handgrip maximum (kg) – mean points left, right	45–55	27–33	
SUE2: Biceps (n)	19–23	17–21	
**Strength lower extremity**			(SLE1 + SLE2)/2
SLE1: 30-sec Chair-stand (n)	18–22	16–20	
SLE2: Single-foot heel rise (n) – mean points left, right	20–25	19–23 right 17–21 left	
**Strength trunk muscles**			(ST1 + ST2 + ST3)/3
ST1: Plank (sec)	84–102	46–56	
ST2: Back Endurance (sec)	88–108	80–98	
ST3: Supine Bridge (sec)	170–207	137–167	
**Mobility**			(M1 + M2 + M3 + M4)/4
M1: Sit-Rise (points)	9–10	10	
M2: Finger-Floor (cm)	0–6	0–6	
M3: Lateral Flexion (cm)	> 17.4	> 16.7	
M4: Beighton Hypermobility Score (points)	0–3 = 0 4–6 = -1 7 − 9 = − 2		
**Balance**			(B1 + B2 + B3 + B4 + B5)/5
B1: SOLEO (sec)	99–121	99–121	
B2: SOLEC (sec)	50–60	50–60	
B3: Functional Reach (cm)	38.3–46.7	33.5–40.9	
B4: Lateral Reach (cm) - mean points left, right	17.1–20.9	17.1–20.9	
B5: Sharpened Romberg (sec)	41–51	41–51	
**Posture**			(Po + Po2 + Po3)/3
Po1: Occiput-to-wall	No = -1 Yes = 0		
Po2: Navicular Drop (within normal limits 5–9 mm) - mean points left, right	No = -1 Yes = 0		
Po3: Patella Mobility - mean points left, right	Yes = -1 No = 0		
**Weight**			(W1 + W2)/2
W1: Body mass index– Overweight (25.0-29.9) = -1 Underweight (< 18.5) = -1 Obesity (> 30.0) = -2	18.5–24.9	18.5–24.9	
W2: Waist circumference	< 94.0	< 80.0	
**Self-assessed physical activity**			
PA1: SGPALS (4 choices)	Physically inactive = -2 Some light physical activity = 0 Regular physical activity and training = 1 Regular hard physical training = 2		(PA1 + PA2 + PA3)/3
PA2: NBHW-PA (minutes exercise/week)*2 + minutes non-strenuous activity	270–329	270–329	
PA3: SED-GIH (hours/day)	< 4 = 2 4–6 = 1 7–9 = 0 10–12 = -1 > 12 =-2		
**Pain**			(P1 + P2 + P3)/3
P1: Pain diagram	No pain areas = 0 1 pain area = -1 > 1 pain area = -2		
P2: ÖMPSQ-SF	0–49 = 0 50–60 = -1 > 60 = -2		
P3: EQ-5D pain question	No pain or discomfort = 0 Moderate pain or discomfort = -1 Extreme pain or discomfort = -2		

*0-levels are found in or extrapolated from references given for each test in Table 2. ÖMPSQ-SF=Örebro Musculoskeletal Pain screening Questionnaire– short form. EQ-5D = Euroqol-5 dimensions

### Feasibility investigation

Of those who received an invitation letter to participate in the study, 7% registered interest and 5% were included for a total of 27 participants. Two people were lost to follow-up because of personal reasons unconnected to the project. Another two were unable to participate in complete examinations at follow-up because of health reasons. Two participants required interpreters and another two spoke only English and not Swedish. Inclusion examinations were made in September-October 2022 and follow-up in January-February 2023. The people included in the study reported high motivation level to make lifestyle changes to improve their health situation, mean 7.37 (SD 2.20) on a 10-point NRS.

Comparisons of participant characteristics to population norms are shown in Table 4. There was a reasonable spread of demographic, socioeconomic and health variables. Education level was higher than in the general population. The difference in civil state can be explained by the high frequency of common-law relationships in Sweden, while the database only reported registered marriages. Participants were generally healthy. Many were slightly overweight. None had diabetes, cardiovascular or lung diseases. One person had hypertension and two reported depression. Mean scores on screening instruments for mental health were below accepted cut-offs for ill-health (SCI-93 22.11 (SD 21.05), HADS-anxiety 6.11 (SD 4.60), HADS-depression 4.15 (SD 3.41)). Three people (11%) were smokers. Overall, the participant group was somewhat under normal/recommended values on the risk profile and far under maximum values (Fig. 1).

**Table 4:** Comparison of study participants to reference values

	Study	Reference value
Sex^a^: Proportion male/female	1.08	1.06
Education^a^: Proportion university/other	2.44	1.10
Activity^a^: Proportion working/not working	12.5	15.76
Income^a^: Proportion high/low^b^	1.08	1
Born^a^: Proportion Sweden/not Sweden	1.45	1.85
Civil state^a^: Proportion cohabitating/single	2.85	0.96^c^
Body mass index (kg/m^2^) (SD)	25.8 (3.8)	< 25.0
Waist circumference male (cm) (SD)	96.6 (8.7)	< 94.0
Waist circumference female (cm) (SD)	84.0 (8.9)	< 80.0
Glucose (mmol/L) (SD)	5.1 (0.7)	4.0–6.0
Total cholesterol (mmol/L) (SD)	4.5 (1.0)	3.3–6.9
Triglycerides (mmol/L) (SD)	1.2 (0.8)	0.45–2.6
LDL/HDL (SD)	2.5 (1.0)	0.4–6.6
Systolic blood pressure (mmHg)	119.1 (12.8)	< 140
Diastolic blood pressure (mmHg)	80.9 (9.1)	< 90

^a^Compared to Swedish population through Statistics Sweden. ^b^Proportion reporting income over Swedish mean for 2022. ^c^Population statistics for Married/not married. SD = Standard deviation

Exertion levels for individual functional tests were in the range 8–16 on the Borg Rating of Perceived Exertion Scale. Mean exertion for the tests was 11.79 (SD 1.42) and exceeded 13 (corresponding to “somewhat hard”) only for the Supine bridge test, the Plank test, the Back endurance test, the 2-minute Step-test and the Single foot heel rises. Participants found the instructions for each test easy to understand– mean 4.86 (SD 0.21) on a 5-point NRS scale.

The participants who required interpreters received some help with the questionnaires from the interpreter, but this took more time than was allotted to the visit. The participants who spoke only English were given English versions of the questionnaires on paper. The examiners were all able to accommodate English speakers. The functional examination took, in most cases, 60–75 min to perform.

Participant feedback expressed on the evaluation questionnaires can be found in Table 5. During the examinations, some participants also gave feedback on the test order and minor adjustments were made during the study (Additional file 2). After conclusion of the intervention, four participants - two who had received lifestyle counselling and two who had not, three female, one male - participated in a focus group concerning their experiences in the study. These are summarised in Table 6.

**Table 5:** Participant feedback from questionnaire

Question	NRS (SD) (*N* = 16)
Average exertion level during examination	2.63 (0.89)
How painful were the tests on average?	1.06 (1.48)
Did the examination take too long?	1.25 (1.53)
How understandable were the test instructions?	4.69 (0.48)
Was the number of questionnaires problematic?	0.81 (1.22)
How interested would you be in regular functional examinations within health care?	4.31 (1.08)
To what degree did you feel that the whole intervention– the examinations, the risk profile and the feedback– were of value to you and your health situation?	4.31 (0.70)

NRS = Numerical Rating Scale (low/not at all/to low extent/no value (0)– high/far too long/to high extent/high value (5))

**Table 6:** Participant experiences from the focus group

Subject	Results
Invitation	Felt tempted to get a health assessment and to get help with lifestyle changes. Did not seem too demanding timewise, could be managed around worktimes. Attractive to participate in research and to contribute to public health. The association with established healthcare providers made the study feel “serious”.
Participation	Felt very positive, was encouraged by friends and colleagues who would have liked to participate. The procedure and visits did not take too much time. It was easy to understand and do what needed to be done. It was “fun” to participate in the functional examination and the examination time went quickly. Flexible examination times were appreciated. Surprised over the number of tests and level of exertion but all tests felt relevant. It felt a little old-fashioned without any digital examinations or specialised equipment but in a nice way. The cycle test was not as demanding as expected. It felt very positive to see improvement in the tests and risk levels, especially in relation to the lifestyle changes made after inclusion. The risk profile and feedback felt like a reward for participating; they were worthwhile on a personal level. It felt useful to see where improvements were needed and new insights into own health were made. To get objective results about personal physical capacity was motivating. Participants who did not receive lifestyle counselling initially would have preferred to be in the counselled group to get feedback and support from the beginning of the study.
Suggestions for improvement	Recommend more information about the planned procedures at the first visit. Recommend feedback and documentation on paper rather than in digital format. Recommend more focus on mental health. Recommend more structured focus on goal attainment and planned lifestyle changes at follow-up. Recommend follow-up at the same time of year as inclusion. Many people are less active in the winter. Follow-up time of 3–4 months was too short to accomplish planned changes of activity and lifestyle. Some people may need more support or supervision to make lifestyle changes than merely feedback on functional capacity.
General comments	Screening with functional examinations may lead to detecting people in need of extensive support. It should be positive in a socioeconomic perspective to prevent the development of ill-health through this type of intervention.

Feedback from collaborating clinical personnel was collected (*N* = 2). They found the intervention instructions easy to understand overall, it was clear how they were to lead individual tests and examinations and it was easy to document the results (Mean 4.90 (SD 0.14). They found that the test order worked well. A detailed study protocol form facilitated documentation of results and simplified for the physiotherapist to lead the examination as planned (Additional file 2).

Blood test, questionnaire, functional test and risk level results, while pertinent to giving individualised feedback, did not specifically affect the feasibility of the intervention and are not presented here.

A structured review of all logistics by the research team after the feasibility study led to proposals of suitable modifications of the study protocol for future studies.

Defined feasibility criteria were fulfilled (Table 7).

**Table 7:** Feasibility criteria

Criterium	Goal	Actual	Goal achievement
Main participant evaluation question (5-point NRS)	> 3	4.31	
Personnel evaluation questions (5-point NRS)	> 3	4.90	
Mean time (min)	< 90	60–75	
Mean exertion (Borg)	< 13	11.79	
Mean understandability (5-point NRS)	> 3	4.86	
Proportion solved practical problems	100%	100%	

## Discussion

A relevant battery of functional tests applicable to inactive 40-year-olds without major health problems was compiled. The tests were understandable, required reasonable exertion, and participants enjoyed performing them. A risk profile was produced which participants found easy to understand. It lifted health aspects which were not always specifically considered earlier and seemed to help motivate to lifestyle changes. All participants had room for improvement on the risk profile. Lifestyle counselling was appreciated and helped guide plans for lifestyle change. The whole intervention fulfilled our feasibility requirements regarding participant and personnel experiences, time, exertion, understandability, and practicalities. Participants saw value in the intervention for their personal health situation and were interested in regular examinations within healthcare. Those participants who registered for the study were well-educated, fairly healthy and highly motivated to make lifestyle changes to improve their health status.

There is an abundance of functional tests and test batteries in the literature. Many were not applicable as they were aimed at younger people who participate in high level sports or at older people who have a high risk for injury and disease [43, 95]. Included tests were chosen based on clinical reasoning concerning appropriate difficulty level for the 40-year-old target group, ascertained validity and reliability, and availability of applicable reference values. The chosen tests worked generally well for the examined 40-year-old population. Cut-offs for the 2-minute step-test had been extrapolated to be applicable to 40-years-old but were likely too low as there was often a considerable difference in point values between this test and the Ekblom-Bak cycle test on the risk profile. This led to perhaps better values for fitness on the risk profile than were warranted. An adjustment of calculation of risk level for fitness is planned before the risk profile is used on other groups. Otherwise, we plan to employ the test protocol and risk profile as they are in future studies.

Grouping test performance according to dimension, in other words all the balance tests together or all the mobility tests together, would have been easier for the physiotherapist leading the functional examination. The test order was, however, determined based on the exertion level for the participant. “Easy” tests were interspersed between more strenuous tests to give time for recovery without wasting examination time (Additional file 2). Self-assessed exertion levels were within reasonable limits with a good spread of strenuous and non-strenuous tests which helped finalise the test order. Written instructions were prepared for each test, but the examiner was allowed to instruct freely, as a physiotherapist might do in a clinical situation. The participants found that this worked well and that they understood what they were supposed to do. REDCap was chosen as a secure electronic case report form. It worked well for data collection from the examination and questionnaires. However, difficulties ensued for non-Swedish speakers, for whom individual solutions needed to be devised [96].

The 40-year-old population was targeted as a possible optimal age for interventions aimed at preventing lifestyle-related disorders based on clinical reasoning and targeted ages in other preventive interventions [97]. Literature has lifted the importance of identifying and utilising “teachable moments” when motivating behavioural change [98]. A new age decade may stimulate thoughts about maintaining health for certain groups. At the age of 40, most people are still generally healthy but may start to notice subtle signs of ageing and reduced functional capacity. While there is no exact age limit for becoming middle-aged, 40-years is one of the accepted cut-offs and may affect how people think of themselves. If we had examined 50-year-olds, it is likely that a somewhat higher proportion would already have established lifestyle-related disorders, while if we chose 30- or 35-year-olds, we hypothesised that interest for future health and perhaps time for self-care might be lower. Our group of 40-year-old participants were, in fact, very interested in lifestyle counselling but it is unknown how much age influenced their interest.

Telephone screening of interested parties’ physical activity levels was somewhat problematic as people seem to have difficulty evaluating how active they are and are uncertain how strenuous moderate physical activity should be. Only in extreme cases, where the person was completely inactive or trained intensely and frequently were they able to adequately account for their activity levels. As people with a self-defined “normal” activity level could reasonably stand to gain from the intervention, only people who could clearly say they exercised intensely and frequently were excluded [99]. We plan to use the Saltin Grimby Physical Activity Scale in future studies to clarify the cut-off level for physical activity when recruiting. All participants, individually and as a group, had some dimensions with room for improvement on the risk profile and participants in the focus group expressed that the choice of tests and the necessary exertion level felt relevant. So, while the most inactive people have the most to gain through the intervention, the group of “somewhat inactive” people also seem to find the intervention advantageous [99]. We also plan to incorporate the accelerometer data in the physical activity arm of the risk profile in future studies. As the participants were fairly unsure of how active they were, the objective accelerometer measures should help them understand their own exertion levels better.

Participants were invited to participate through contact information provided by Statistics Sweden. It was not possible to guarantee an appropriate spread of demographic variables in this way, especially since the response rate was so low. A geographic area including areas with mixed socioeconomic conditions was chosen but still people with higher education were overrepresented in the study, while other demographic aspects were more congruent with Swedish norms [96, 100]. Civil state is not assessed to be different from populations norms as co-habitation is very common in Sweden, while population statistics reflect only marital status. So, it is primarily educational level which affects generalisability of the results. People with higher education may have areas of employment with more time flexibility. It is possible that the timing of the examinations and number of visits was easier to accommodate for people who had flexible worktimes. Perhaps minimising the number of visits and including examination times outside normal work hours could encourage participation from people with a broader spectrum of educational backgrounds [96, 100]. To reach a participant group with more varied sociodemographic backgrounds, we propose to recruit participants through primary care clinics in a larger study. Each Swedish citizen is registered at a primary care clinic which is responsible for first-line healthcare. By recruiting clinics in geographical areas with varying socioeconomic conditions and contacting their registered 40-year-olds, we hope to increase participation in underrepresented groups. Results from the focus group also point out that connection with known healthcare providers increased credibility for the project organisation and motivation to make contact and register for the study.

Those who were interested in participating in the study were self-assessed as very motivated to making necessary changes in lifestyle. However, only 7% of those who received invitation letters made contact to express interest in participating. Motivation for lifestyle change, unclear self-assessment as to physical activity level, as well as practical difficulties may be among the reasons for the low interest rate. Interest in taking responsibility for maintaining health may generally be higher among people with higher education, such as those who volunteered for this study [101]. However, the uniform high level of motivation for lifestyle changes among all participants restricts the generalisability to highly motivated people. As with many lifestyle interventions, those who are in greatest need of making changes may be the most difficult to reach [101]. However, reaching those who are already motivated to make lifestyle changes but who need knowledge and support to succeed may be more efficient than laying extensive healthcare resources on people who need to make lifestyle changes for health reasons but who are not motivated to do so. This latter group may have greater need of “primordial prevention” aimed at health behaviour on the societal level than the type of primary prevention aimed at risk factors which is investigated in this study [102, 103].

The participants were engaged in the study and felt the intervention was helpful to them on a personal level. Some were surprised by their results. Others got results they expected but felt it helpful to clarify which dimensions they needed to focus on to improve current health or reduce risk for future health problems. It is important to create target group-friendly interventions, such as this one, that awaken interest for preventing health disorders, if we are to succeed in reducing lifestyle-related ill-health.

## Conclusions

This project developed a functional test battery and risk profile aimed at inactive 40-year-olds which fulfilled our feasibility criteria. Functional screening and lifestyle counselling were found to be of value to a sub-group of inactive 40-year-olds who already were motivated to improve their health situations. Attempts will be made in future studies to reach a broader target group.

### Electronic supplementary material

Below is the link to the electronic supplementary material.

Supplementary Material 1

Supplementary Material 2

## Data Availability

The datasets used and/or analysed during the current study are available from the corresponding author on reasonable request.

## Abbreviations

HADS: Hospital Anxiety and depression Scale
LRD: Lifestyle-related Disorders
NBHW: (Swedish) National Board of Health and Welfare
NRS: Numerical Rating Scale
PAP: Physical Activity on Prescription
SCI-93: Stress and Crisis Inventory-93
SD: Standard deviation
VIP: Västerbotten Intervention Programme

## References

[1] Lewis R, Gomez Alvarez CB, Rayman M, Lanham-New S, Woolf A, Mobasheri A (2019). Strategies for optimising musculoskeletal health in the 21(St) century. BMC Musculoskelet Disord.

[2] Guthold R, Stevens GA, Riley LM, Bull FC (2018). Worldwide trends in insufficient physical activity from 2001 to 2016: a pooled analysis of 358 population-based surveys with 1·9 million participants. Lancet Global Health.

[3] Life expectancy and disease burden in the (2019). Nordic countries: results from the Global Burden of diseases, injuries, and risk factors study 2017. Lancet Public Health.

[4] Crosland P, Ananthapavan J, Davison J, Lambert M, Carter R (2019). The economic cost of preventable disease in Australia: a systematic review of estimates and methods. Aust N Z J Public Health.

[5] Schäfer I, Hansen H, Ruppel T, Lühmann D, Wagner HO, Kazek A (2020). Regional differences in reasons for consultation and general practitioners’ spectrum of services in northern Germany - results of a cross-sectional observational study. BMC Fam Pract.

[6] Jallinoja P, Absetz P, Kuronen R, Nissinen A, Talja M, Uutela A (2007). The dilemma of patient responsibility for lifestyle change: perceptions among primary care physicians and nurses. Scand J Prim Health Care.

[7] Korczak D, Dietl M, Steinhauser G (2011). Effectiveness of programmes as part of primary prevention demonstrated on the example of cardiovascular diseases and the metabolic syndrome. GMS Health Technol Assess.

[8] Blomstedt Y, Norberg M, Stenlund H, Nyström L, Lönnberg G, Boman K (2015). Impact of a combined community and primary care prevention strategy on all-cause and cardiovascular mortality: a cohort analysis based on 1 million person-years of follow-up in Västerbotten County, Sweden, during 1990–2006. BMJ Open.

[9] Virtanen M, Ervasti J, Head J, Oksanen T, Salo P, Pentti J (2018). Lifestyle factors and risk of sickness absence from work: a multicohort study. Lancet Public Health.

[10] Ayis S, Dieppe P (2009). The natural history of disability and its determinants in adults with lower limb musculoskeletal pain. J Rheumatol.

[11] Puthoff M. Participants’ perceptions and the implementation of a physical fitness screen for aging adults. J Geriatr Phys Ther. 2019. 10.1519/JPT.0000000000000241 31373944

[12] Merder-Coşkun D, Uzuner A, Keniş-Coşkun Ö, Çelenlioğlu AE, Akman M, Karadağ-Saygı E (2017). Relationship between obesity and musculoskeletal system findings among children and adolescents. Turkish J Phys Med Rehabilitation.

[13] WHO. Global recommendations on physical activity for health: World Health Organization; 2011 [2020-09-25]. Available from: https://www.who.int/dietphysicalactivity/factsheet_adults/en/26180873

[14] Onerup A, Arvidsson D, Blomqvist Å, Daxberg E-L, Jivegård L, Jonsdottir IH (2019). Physical activity on prescription in accordance with the Swedish model increases physical activity: a systematic review. Br J Sports Med.

[15] Atienza AA, Moser RP, Perna F, Dodd K, Ballard-Barbash R, Troiano RP (2011). Self-reported and objectively measured activity related to biomarkers using NHANES. Med Sci Sports Exerc.

[16] Dewi TK, Massar K, Ruiter RAC, Leonardi T (2019). Determinants of breast self-examination practice among women in Surabaya, Indonesia: an application of the health belief model. BMC Public Health.

[17] Rosenstock IM (1966). Why people use health services. Milbank Mem Fund Q.

[18] Docherty B, Sheridan N, Kenealy T (2016). Developing brief opportunistic interactions: practitioners facilitate patients to identify and change health risk behaviours at an early preventive stage. Prim Health Care Res Dev.

[19] Miller SJ, Foran-Tuller K, Ledergerber J, Jandorf L (2017). Motivational interviewing to improve health screening uptake: a systematic review. Patient Educ Couns.

[20] Lee WW, Choi KC, Yum RW, Yu DS, Chair SY (2016). Effectiveness of motivational interviewing on lifestyle modification and health outcomes of clients at risk or diagnosed with cardiovascular diseases: a systematic review. Int J Nurs Stud.

[21] McGrane N, Galvin R, Cusack T, Stokes E (2015). Addition of motivational interventions to exercise and traditional physiotherapy: a review and meta-analysis. Physiotherapy.

[22] Ervin RB. Prevalence of metabolic syndrome among adults 20 years of age and over, by sex, age, race and ethnicity, and body mass index: United States, 2003–2006. Natl Health Stat Rep. 2009;(13):1–7. 19634296

[23] Norberg M, Wall S, Boman K, Weinehall L (2010). The Västerbotten intervention Programme: background, design and implications. Global Health Action.

[24] Harris PA, Taylor R, Thielke R, Payne J, Gonzalez N, Conde JG (2009). Research electronic data capture (REDCap)—A metadata-driven methodology and workflow process for providing translational research informatics support. J Biomed Inform.

[25] Harris PA, Taylor R, Minor BL, Elliott V, Fernandez M, O’Neal L (2019). The REDCap consortium: building an international community of software platform partners. J Biomed Inform.

[26] Ericsson A, Nyström C, Mannerkorpi K (2015). Psychometric properties of the stress and Crisis Inventory (SCI-93) in females with fibromyalgia and chronic widespread pain. Nord J Psychiatry.

[27] Bjelland I, Dahl AA, Haug TT, Neckelmann D (2002). The validity of the hospital anxiety and Depression Scale: an updated literature review. J Psychosom Res.

[28] Grimby G, Börjesson M, Jonsdottir IH, Schnohr P, Thelle DS, Saltin B (2015). The saltin-Grimby Physical Activity Level Scale and its application to health research. Scand J Med Sci Sports.

[29] Ekblom Ö, Ekblom-Bak E, Bolam KA, Ekblom B, Schmidt C, Söderberg S (2015). Concurrent and predictive validity of physical activity measurement items commonly used in clinical settings–data from SCAPIS pilot study. BMC Public Health.

[30] Rosengren A, Wilhelmsen L (1997). Physical activity protects against coronary death and deaths from all causes in middle-aged men. Evidence from a 20-year follow-up of the primary prevention study in Göteborg. Ann Epidemiol.

[31] Olsson SJ, Ekblom Ö, Andersson E, Börjesson M, Kallings LV (2016). Categorical answer modes provide superior validity to open answers when asking for level of physical activity: a cross-sectional study. Scand J Public Health.

[32] Ek A, Kallings LV, Ekström M, Börjesson M, Ekblom Ö. Subjective reports of physical activity levels and sedentary time prior to hospital admission can predict utilization of hospital care and all-cause mortality among patients with cardiovascular disease. Eur J Cardiovasc Nurs. 2020:1474515120921986. 10.1177/1474515120921986 PMC7817990 32370681

[33] Kallings LV, Olsson SJG, Ekblom Ö, Ekblom-Bak E, Börjesson M. The SED-GIH: a single-item question for assessment of stationary behavior-a study of concurrent and convergent validity. Int J Environ Res Public Health. 2019;16(23). 10.3390/ijerph16234766 PMC6926785 31795109

[34] Hurst NP, Kind P, Ruta D, Hunter M, Stubbings A (1997). Measuring health-related quality of life in rheumatoid arthritis: validity, responsiveness and reliability of EuroQol (EQ-5D). Br J Rheumatol.

[35] Linton SJ, Hallden K (1998). Can we screen for problematic back pain? A screening questionnaire for predicting outcome in acute and subacute back pain. Clin J Pain.

[36] Linton SJ, Boersma K (2003). Early identification of patients at risk of developing a persistent back problem: the predictive validity of the Orebro Musculoskeletal Pain Questionnaire. Clin J Pain.

[37] Hockings RL, McAuley JH, Maher CG (2008). A systematic review of the predictive ability of the Orebro Musculoskeletal Pain Questionnaire. Spine (Phila Pa 1976).

[38] Borg G (1977). Physical work and effort.

[39] Björkman F, Ekblom-Bak E, Ekblom Ö, Ekblom B (2016). Validity of the revised Ekblom Bak cycle ergometer test in adults. Eur J Appl Physiol.

[40] GIH. Referensvärden för maximal syreupptagning vid genomförande av Ekblom Bak-test. Stockholm: Gymnastik- och idrottshögskolan/ The Swedish School of Sport and Health Sciences; 2017 [updated 2017-08-01; cited 2020 2020-09-21]. Available from: https://www.gih.se/ekblombaktest

[41] Rikli RE, Jones CJ (2013). Development and validation of criterion-referenced clinically relevant fitness standards for maintaining physical independence in later years. Gerontologist.

[42] Rikli RE, Jones CJ (1999). Functional fitness normative scores for community-residing older adults, ages 60–94. J Aging Phys Activity.

[43] Rikli RE, Jones CJ (1999). Development and validation of a functional fitness test for community-residing older adults. J Aging Phys Activity.

[44] Freene N, Waddington G, Davey R, Cochrane T (2015). Longitudinal comparison of a physiotherapist-led, home-based and group-based program for increasing physical activity in community-dwelling middle-aged adults. Aust J Prim Health.

[45] Bohannon RW, Crouch RH (2019). Two-minute step test of Exercise Capacity: systematic review of procedures, performance, and Clinimetric Properties. J Geriatr Phys Ther.

[46] Leong DP, Teo KK, Rangarajan S, Kutty VR, Lanas F, Hui C (2016). Reference ranges of handgrip strength from 125,462 healthy adults in 21 countries: a prospective urban rural epidemiologic (PURE) study. J Cachexia Sarcopenia Muscle.

[47] Bohannon RW, Wang YC, Yen SC, Grogan KA, Handgrip Strength (2019). A comparison of values obtained from the NHANES and NIH Toolbox Studies. Am J Occup Ther.

[48] Gerodimos V (2012). Reliability of handgrip strength test in basketball players. J Hum Kinetics.

[49] Savva C, Giakas G, Efstathiou M, Karagiannis C (2014). Test-retest reliability of handgrip strength measurement using a hydraulic hand dynamometer in patients with cervical radiculopathy. J Manipulative Physiol Ther.

[50] Leong DP, Teo KK, Rangarajan S, Lopez-Jaramillo P, Avezum A Jr., Orlandini A et al. Prognostic value of grip strength: findings from the prospective urban rural epidemiology (PURE) study. Lancet. 2015. 10.1016/S0140-6736(14)62000-6 25982160

[51] Bohannon RW (2019). Grip strength: an indispensable biomarker for older adults. Clin Interv Aging.

[52] Gurses HN, Zeren M, Denizoglu Kulli H, Durgut E (2018). The relationship of sit-to-stand tests with 6-minute walk test in healthy young adults. Med (Baltim).

[53] Jones CJ, Rikli RE, Beam WC (1999). A 30-s chair-stand test as a measure of lower body strength in community-residing older adults. Res Q Exerc Sport.

[54] Hébert-Losier K, Wessman C, Alricsson M, Svantesson U (2017). Updated reliability and normative values for the standing heel-rise test in healthy adults. Physiotherapy.

[55] Lunsford BR, Perry J (1995). The standing heel-rise test for ankle plantar flexion: criterion for normal. Phys Ther.

[56] Vårbakken K, Lorås H, Nilsson KG, Engdal M, Stensdotter AK (2019). Relative difference in muscle strength between patients with knee osteoarthritis and healthy controls when tested bilaterally and joint-inclusive: an exploratory cross-sectional study. BMC Musculoskelet Disord.

[57] Schellenberg KL, Lang JM, Chan KM, Burnham RS (2007). A clinical tool for office assessment of lumbar spine stabilization endurance: prone and supine bridge maneuvers. Am J Phys Med Rehabil.

[58] De Blaiser C, De Ridder R, Willems T, Danneels L, Vanden Bossche L, Palmans T (2018). Evaluating abdominal core muscle fatigue: Assessment of the validity and reliability of the prone bridging test. Scand J Med Sci Sports.

[59] Vanti C, Conti C, Faresin F, Ferrari S, Piccarreta R (2016). The relationship between clinical instability and endurance tests, Pain, and disability in nonspecific low back Pain. J Manipulative Physiol Ther.

[60] Biering-Sørensen F (1984). Physical measurements as risk indicators for low-back trouble over a one-year period. Spine.

[61] Latimer J, Maher CG, Refshauge K, Colaco I (1999). The reliability and validity of the Biering-Sorensen test in asymptomatic subjects and subjects reporting current or previous nonspecific low back pain. Spine (Phila Pa 1976).

[62] Coorevits P, Danneels L, Cambier D, Ramon H, Vanderstraeten G (2008). Assessment of the validity of the Biering-Sørensen test for measuring back muscle fatigue based on EMG median frequency characteristics of back and hip muscles. J Electromyogr Kinesiol.

[63] Alaranta H, Hurri H, Heliövaara M, Soukka A, Harju R (1994). Non-dynamometric trunk performance tests: reliability and normative data. Scand J Rehabil Med.

[64] Araújo CGS, Castro CLB, Franca JFC, Araújo DS (2020). Sitting–rising test: sex- and age-reference scores derived from 6141 adults. Eur J Prev Cardiol.

[65] Brito LB, de Araújo DS, de Araújo CG (2013). Does flexibility influence the ability to sit and rise from the floor?. Am J Phys Med Rehabil.

[66] Brito LB, Ricardo DR, Araújo DS, Ramos PS, Myers J, Araújo CG (2014). Ability to sit and rise from the floor as a predictor of all-cause mortality. Eur J Prev Cardiol.

[67] Michel A, Kohlmann T, Raspe H (1997). The association between clinical findings on physical examination and self-reported severity in back pain. Results of a population-based study. Spine (Phila Pa 1976).

[68] Nicolaisen T, Jørgensen K (1985). Trunk strength, back muscle endurance and low-back trouble. Scand J Rehabil Med.

[69] Cougot B, Petit A, Paget C, Roedlich C, Fleury-Bahi G, Fouquet M (2015). Chronic low back pain among French healthcare workers and prognostic factors of return to work (RTW): a non-randomized controlled trial. J Occup Med Toxicol.

[70] Mannion AF, Caporaso F, Pulkovski N, Sprott H (2012). Spine stabilisation exercises in the treatment of chronic low back pain: a good clinical outcome is not associated with improved abdominal muscle function. Eur Spine J.

[71] Wakhlu A, Chandran V, Phumethum V, Shen H, Cook RJ, Gladman D (2019). Comparison between INSPIRE and Domjan method for measuring lumbar lateral flexion in patients of psoriatic arthritis (PsA) and correlation with radiographic damage. Clin Rheumatol.

[72] Domján L, Nemes T, Bálint GP, Tóth Z, Gömör B (1990). A simple method for measuring lateral flexion of the dorsolumbar spine. J Rheumatol.

[73] Sadler SG, Spink MJ, Ho A, De Jonge XJ, Chuter VH (2017). Restriction in lateral bending range of motion, lumbar lordosis, and hamstring flexibility predicts the development of low back pain: a systematic review of prospective cohort studies. BMC Musculoskelet Disord.

[74] Adams MA, Mannion AF, Dolan P (1999). Personal risk factors for first-time low back pain. Spine (Phila Pa 1976).

[75] Alaranta H, Hurri H, Heliövaara M, Soukka A, Harju R (1994). Flexibility of the spine: normative values of goniometric and tape measurements. Scand J Rehabil Med.

[76] Kumar B, Lenert P (2017). Joint hypermobility syndrome: recognizing a commonly overlooked cause of Chronic Pain. Am J Med.

[77] Engelbert RHH, Juul-Kristensen B, Pacey V, de Wandele I, Smeenk S, Woinarosky N (2017). The evidence-based rationale for physical therapy treatment of children, adolescents, and adults diagnosed with joint hypermobility syndrome/hypermobile Ehlers Danlos syndrome. American journal of medical genetics part C. Seminars Med Genet.

[78] Tidstrand J, Horneij E (2009). Inter-rater reliability of three standardized functional tests in patients with low back pain. BMC Musculoskelet Disord.

[79] Morioka S, Fukumoto T, Hiyamizu M, Matsuo A, Takebayashi H, Miyamoto K (2012). Changes in the equilibrium of standing on one leg at various life stages. Curr Gerontol Geriatr Res.

[80] Duncan PW, Studenski S, Chandler J, Prescott B (1992). Functional reach: predictive validity in a sample of elderly male veterans. J Gerontol.

[81] Isles RC, Choy NLL, Steer M, Nitz JC (2004). Normal values of balance tests in women aged 20–80. J Am Geriatr Soc.

[82] Holbein-Jenny MA, Billek-Sawhney B, Beckman E, Smith T (2005). Balance in personal care home residents: a comparison of the Berg Balance Scale, the multi-directional Reach Test, and the activities-specific balance confidence scale. J Geriatr Phys Ther.

[83] Smith PS, Hembree JA, Thompson ME (2004). Berg Balance Scale and Functional Reach: determining the best clinical tool for individuals post acute stroke. Clin Rehabil.

[84] Duncan PW, Weiner DK, Chandler J, Studenski S (1990). Functional reach: a new clinical measure of balance. J Gerontol.

[85] Weiner DK, Duncan PW, Chandler J, Studenski SA (1992). Functional reach: a marker of physical frailty. J Am Geriatr Soc.

[86] Brauer S, Burns Y, Galley P (1999). Lateral reach: a clinical measure of medio-lateral postural stability. Physiother Res Int.

[87] El-Kashlan HK, Shepard NT, Asher AM, Smith-Wheelock M, Telian SA (1998). Evaluation of clinical measures of equilibrium. Laryngoscope.

[88] Franchignoni F, Tesio L, Martino MT, Ricupero C (1998). Reliability of four simple, quantitative tests of balance and mobility in healthy elderly females. Aging (Milano).

[89] Bohannon RW, Tudini F, Constantine D (2019). Tragus-to-wall: a systematic review of procedures, measurements obtained, and clinimetric properties. J Back Musculoskelet Rehabil.

[90] Menz HB, Dufour AB, Riskowski JL, Hillstrom HJ, Hannan MT (2013). Foot posture, foot function and low back pain: the Framingham Foot Study. Rheumatology (Oxford).

[91] Zuil-Escobar JC, Martínez-Cepa CB, Martín-Urrialde JA, Gómez-Conesa A (2018). Medial longitudinal Arch: Accuracy, Reliability, and correlation between Navicular Drop Test and Footprint parameters. J Manipulative Physiol Ther.

[92] Cote KP, Brunet ME, Gansneder BM, Shultz SJ (2005). Effects of Pronated and Supinated Foot postures on Static and Dynamic Postural Stability. J Athl Train.

[93] Li JS, Tsai TY, Felson DT, Li G, Lewis CL (2017). Six degree-of-freedom knee joint kinematics in obese individuals with knee pain during gait. PLoS ONE.

[94] Onate JA, Everhart JS, Clifton DR, Best TM, Borchers JR, Chaudhari AM (2016). Physical exam risk factors for Lower Extremity Injury in High School athletes: a systematic review. Clin J Sport Med.

[95] Bonazza NA, Smuin D, Onks CA, Silvis ML, Dhawan A (2017). Reliability, validity, and Injury Predictive Value of the Functional Movement screen: a systematic review and Meta-analysis. Am J Sports Med.

[96] Davis TC, Arnold CL, Mills G, Miele L (2019). A qualitative study exploring barriers and facilitators of enrolling underrepresented populations in clinical trials and biobanking. Front cell Dev Biology.

[97] Sveriges_Regioner_i_Samverkan. Slutrapport avseende systematisk kunskapsgenomgång av den svenska modellen för Riktade hälsosamtal. NPO Levnadsvanor - Nationellt system för kunskapsstyrning Hälso- och sjukvård; 2022.

[98] Lawson PJ, Flocke SA (2009). Teachable moments for health behavior change: a concept analysis. Patient Educ Couns.

[99] Cunningham C, O’ Sullivan R, Caserotti P, Tully MA (2020). Consequences of physical inactivity in older adults: a systematic review of reviews and meta-analyses. Scand J Med Sci Sports.

[100] Pampel FC, Krueger PM, Denney JT (2010). Socioeconomic disparities in Health behaviors. Annu Rev Sociol.

[101] Lindgren M, Börjesson M, Ekblom Ö, Bergström G, Lappas G, Rosengren A (2016). Physical activity pattern, cardiorespiratory fitness, and socioeconomic status in the SCAPIS pilot trial - A cross-sectional study. Prev Med Rep.

[102] Vaduganathan M, Venkataramani AS, Bhatt DL (2015). Moving toward Global Primordial Prevention in Cardiovascular Disease: the heart of the Matter. J Am Coll Cardiol.

[103] Greenberg H (2022). An inflection point in global public health. Globalization Health.

