# Supplementary material for: Development and feasibility of a function-based preventive intervention for lifestyle-related disorders
Source: BMC Public Health. 2024 Mar 4;24:681. doi: 10.1186/s12889-024-18017-8 (PMC10910714; doi:10.1186/s12889-024-18017-8)
Supplement: Supplementary file 1 — Supplementary Material 1 [file 12889_2024_18017_MOESM1_ESM.docx]

Project-specific questionnaires:

**Smoking habits**

How would you best describe your smoking habits?

Never been a smoker ____
Stopped smoking more than 6 months ago ­­____
Stopped smoking less than 6 months ago ____
Smoke but not daily ____
Daily smokers (number of cigarettes/day on a normal day):
 1-9 ____
 10-19 ____
 20 or more ____

**Self-estimated physical function and motivation level**

**Here come a few questions about how you estimate your physical functional capacity and your level of motivation for change during the latest month:**

Place an **X** in the box that best describes how you estimate your capacity for the following functions:

|  | Very bad | Bad | Neither good nor bad | Good | Very good | Don’t know |
| --- | --- | --- | --- | --- | --- | --- |
| Strength in hands and arms |  |  |  |  |  |  |
| Strength in legs |  |  |  |  |  |  |
| Balance |  |  |  |  |  |  |
| Fitness* |  |  |  |  |  |  |

*Ability to exert yourself for a sustained period

Do you think you can walk 5 km (1 hour)?

No

With much effort/difficulty

With some effort/difficulty

With a little effort/difficulty

Without problem

How motivated are you to make lifestyle changes to improve your health?

0 1 2 3 4 5 6 7 8 9 10
Not at all motivated Very strongly motivated
