# Supplementary material for: Development and feasibility of a function-based preventive intervention for lifestyle-related disorders
Source: BMC Public Health. 2024 Mar 4;24:681. doi: 10.1186/s12889-024-18017-8 (PMC10910714; doi:10.1186/s12889-024-18017-8)
Supplement: Supplementary file 2 — Supplementary Material 2 [file 12889_2024_18017_MOESM2_ESM.docx]

Supplementary Table 1. Test order for functional examination and documentation protocol

|  | **Test** |  | **Results** |  | **Results** |  |
| --- | --- | --- | --- | --- | --- | --- |
| 1 | Ekblom-Bak | Separate documentation | |  |  |  |
| 2 | Knees - locked? | Right | Yes/no | **SHOES OFF** |  |  |
|  |  | Left | Yes/no |  |  |  |
| 3 | Navicular drop | Right-unloaded |  |  |  |  |
|  |  | Right-loaded |  |  |  |  |
|  |  | Left-unloaded |  |  |  |  |
|  |  | Left-loaded |  |  |  |  |
| 4 | Handgrip strength | Right 1 |  | Left 1 |  |  |
|  |  | Right 2 |  | Left 2 |  |  |
|  |  | Right 3 |  | Left 3 |  |  |
| 5 | Supine Bridge | Time |  | *2 min + stretch dominant* | |  |
| 6 | SOLEO - time | R/L |  |  |  |  |
| 7 | Biceps - number | R/L |  |  |  |  |
| 8 | SOLEC - time | R/L |  |  |  |  |
| 9 | Plank | Time |  |  |  |  |
| 10 | Beighton | Dig V | R/L |  |  |  |
|  |  | Wrist | R/L |  |  |  |
|  |  | Elbow | R/L |  |  |  |
|  |  | Knees | R/L |  |  |  |
|  |  | Back |  |  |  |  |
| 11 | Fingertip-floor | Distance |  |  |  |  |
| 12 | Sit-rise - points | Sit 5p |  | Sit 5p |  |  |
|  |  | Rise 5p |  | Rise 5p |  |  |
| 13 | 2-min Step-test | Number |  | **SHOES ON** |  |  |
| 14 | Occiput-to-wall | Contact? | Yes/no |  |  |  |
| 15 | Functional reach | Attempt 1 |  | Attempt 2 |  |  |
| 16 | Heel rises - number | Right |  | Left |  |  |
| 17 | Lateral reach | Right 1 |  | Left 1 |  |  |
|  |  | Right 2 |  | Left 2 |  |  |
|  |  | Right 3 |  | Left 3 |  |  |
| 18 | Sharpened Romberg | Attempt 1 |  |  |  |  |
|  |  | Attempt 2 |  |  |  |  |
|  |  | Attempt 3 |  |  |  |  |
| 19 | Back extension | Time |  |  |  |  |
|  |  | | | | | Distance |
| 20 | Lateral flexion | Right 1 |  | Right 2 |  |  |
|  |  | Left 1 |  | Left 2 |  |  |
| 21 | Chair-stand | Number |  |  |  |  |
